# Role of the APOE ε2/ε3/ε4 Polymorphism in the Development of Primary Open-Angle Glaucoma: Evidence from a Comprehensive Meta-Analysis

**DOI:** 10.1371/journal.pone.0082347

**Published:** 2013-11-27

**Authors:** Qinglu Song, Pin Chen, Qinghuai Liu

**Affiliations:** 1 Department of Ophthalmology, the First Affiliated Hospital of Nanjing Medical University, Nanjing, P.R. China; 2 Department of Neurosurgery, the First Affiliated Hospital of Nanjing Medical University, Nanjing, P.R. China; Massachusetts Eye & Ear Infirmary, Harvard Medical School, United States of America

## Abstract

Primary open-angle glaucoma (POAG) is one of the leading causes of blindness worldwide. The association between the APOE ε2/ε3/ε4 polymorphism and the risk of POAG has been widely reported, but the results of previous studies remain controversial. To comprehensively evaluate the APOE ɛ2/ɛ3/ε4 polymorphism on the genetic risk for POAG, we performed a systematic review and meta-analysis of previously published studies. The PubMed and Web of Science databases were systematically searched to identify relevant studies. Data were extracted from these studies and odds ratios with corresponding 95% confidence intervals were computed to estimate the strength of the association. Stratified analyses according to ethnicity and sensitivity analyses were also conducted for further confirmation. A total of nine studies were eligible for the meta-analysis, and these studies included data on 1928 POAG cases and 1793 unrelated match controls. The combined results showed that there were no associations between the APOE ε2/ε3/ε4 polymorphism and POAG risk in any of the 10 comparison models. The analysis that was stratified by ethnicity subgroups also failed to reveal a significant association. The sensitivity analysis confirmed the stability and reliability of the findings. There was no risk of publication bias. Our meta-analysis provides strong evidence that the APOE ε2/ε3/ε4 polymorphism is not associated with POAG susceptibility in any populations.

## Introduction

Glaucoma is defined as a multifactorial optic neuropathy that is characterized by a progressive loss of retinal ganglion cells in the optic disc or retinal nerve fiber [1]. Glaucoma is the leading cause of irreversible blindness worldwide and has become one of the most challenging health issues currently being confronted by mankind [2]. Primary open angle glaucoma (POAG) is the most prevalent subtype of glaucoma and causes approximately 3.3 million cases of bilateral blindness worldwide [3,4]. Currently, the diagnosis of POAG still relies on a combination of tests that measure intraocular pressure (IOP), test for decreased automated visual field function and identify increases in the optic cup-to-disc ratio. Despite its characteristic clinical features and distinctive pathogenesis, the detailed etiology of POAG has not been fully elucidated. It is widely accepted that multiple factors including genetic and environmental factors, and their interactions, likely contribute to the development of POAG [5-7]. Currently, several genes have been reported to be associated with POAG, and the apolipoprotein E (APOE) gene has received increasing attention [8-12].

APOE is the major apolipoprotein of the central nervous system and plays important roles in several biological processes and in neural function [13]. APOE is essential for normal lipoprotein transformation and metabolic processes [14,15]. An in vivo study of rabbit retinas revealed that the APOE protein is synthesized by Müller cells (the predominant glial cells of the retina), absorbed by the ganglion cells, and plays an important role in axonal nutrition [16]. 

The APOE gene has been mapped to the 19q13 region, and its common polymorphism has three alleles in exon 4, namely, ε2, ε3, and ε4. These three alleles define the following six APOE phenotypes:ε2/ε2, ε3/ε3, ε4/ε4, ε2/ε3, ε2/ε4, and ε3/ε4 [17]. The APOE ε3 allele is the most (77%) common, and ε2 allele is the least (8%) common. The frequency of the APOE ε4 allele is approximately 15% in the general population [18-20]. It is thought that certain variant genotypes of the APOE gene may encode different types of protein that differ significantly in structure and functions [21,22].

In the past decade, a great number of molecular epidemiological studies have been performed to evaluate the association between APOE gene polymorphisms and POAG susceptibility in diverse populations, but results are somewhat controversial and underpowered likely because of the limitations of limitation of individual studies. Therefore, we carried out this meta-analysis to determine whether this polymorphism is associated with the risk of APOE by collecting and sorting previously published studies.

## Materials and Methods

### Identification and eligibility of relevant studies

Systematic literature searched of the Pubmed and Web of Science databases were performed to identify relevant studies that have investigated the association between POAG risk and the APOE ε2/ε3/ε4 polymorphism using the following search terms: “apolipoprotein E or APOE” and “glaucoma or POAG” (up to July 20, 2013). Only those published in the English language with available full text articles were included. All studies return by these searches were retrieved, and the references in these articles were also checked for additional relevant published articles. For studies with overlapping data that were published by same investigators, only the most recent or complete study was included. Meeting abstracts, case reports, editorials, review articles, and letters were excluded. Studies included in the current meta-analysis had to meet the following criteria: (1) contain evaluations of the APOE ε2/ε3/ε4 polymorphism and POAG risk, (2) have an unrelated case-control design, and (3) have sufficient data (i.e., genotype distributions for the cases and controls) to estimate an odds ratio (OR) and its 95%CI. Major reasons for the exclusion of studies included a lack of POAG research, a lack of a control population, duplication of previous publications and unavailable genotype frequencies. 

### Data extraction

Two separate investigators reviewed and extracted data from all eligible publications independently based on the inclusion and exclusion criteria listed above. Discrepancies were adjudicated by a third reviewer until consensus was reached on all items. The following variables were extracted from each study if available: the first author’s surname, the year of publication, the country of origin, the ethnicity of the study samples, the numbers of cases and controls, and the numbers of cases and controls with different genotypes. Ethnic backgrounds were categorized as Caucasian, Asian or Other (i.e., not Caucasian or Asian). 

### Statistical analyses

All statistical tests in the current meta-analysis were conducted using Stata (Version 11.0, Stata Corporation). All P-values were two-sided, and p<0.05 was considered statistically significant. The strength of the association between the APOE ε2/ε3/ε4 polymorphism and the risk of APOE was measured using odds ratios (ORs) with 95% confidence intervals (CIs). In our meta-analysis the ε3/ε3 genotype and ε3 allele variant were used as the reference group. We examined the association between genetic variants (both ε2 and ε4 variation) and POAG risk with an allelic comparison model (ε2 vs ε3 or ε4 vs ε3), and made comparisons with homozygotes (ε2/2 vs ε3/3 or ε4/4 vs ε3/3), heterozygotes (ε2/3 vs ε3/3 or ε3/4 vs ε3/3), the dominant genetic model (ε2/2 + ε2/3 vs ε3/3 or ε3/4 + ε4/4 vs ε3/3), and the recessive genetic model (ε2/2 vs ε3/3+ ε2/3 or ε4/4 vs ε3/3 + ɛ3/4). Subgroup analyses were also performed by ethnicity.

The existence of heterogeneity between studies was ascertained with the Chi-square-based Q statistic. The Q-test yielding P values greater than 0.05 indicated a lack of heterogeneity between studies and resulted in the pooled OR estimate of each study being calculated using a fixed-effects model (the Mantel–Haenszel method) [23]; otherwise, a random-effects model (the DerSimonian and Laird method) was used [24]. The I^2^ statistic was then used to quantify the proportion of the total variation that was due to heterogeneity; I^2^<25%, 25–75% and >75% represented low, moderate or high degrees of inconsistency, respectively [25,26].

Hardy-Weinberg equilibriums (HWEs) were calculated with goodness-of-fit tests (i.e., chi-square or Fisher’s exact tests). One-way sensitivity analyses were carried out by consecutively omitting one study at a time to assess the stability of the meta-analysis results. Visual inspection of the asymmetry of funnel plots was carried out to assess potential publication bias. Begg’s funnel plots and test were used to statistically estimate publication bias (P<0.05 was taken to indicate significant publication bias) [27]. 

## Results

### Main characteristics of all of the available studies

**Figure 1:**
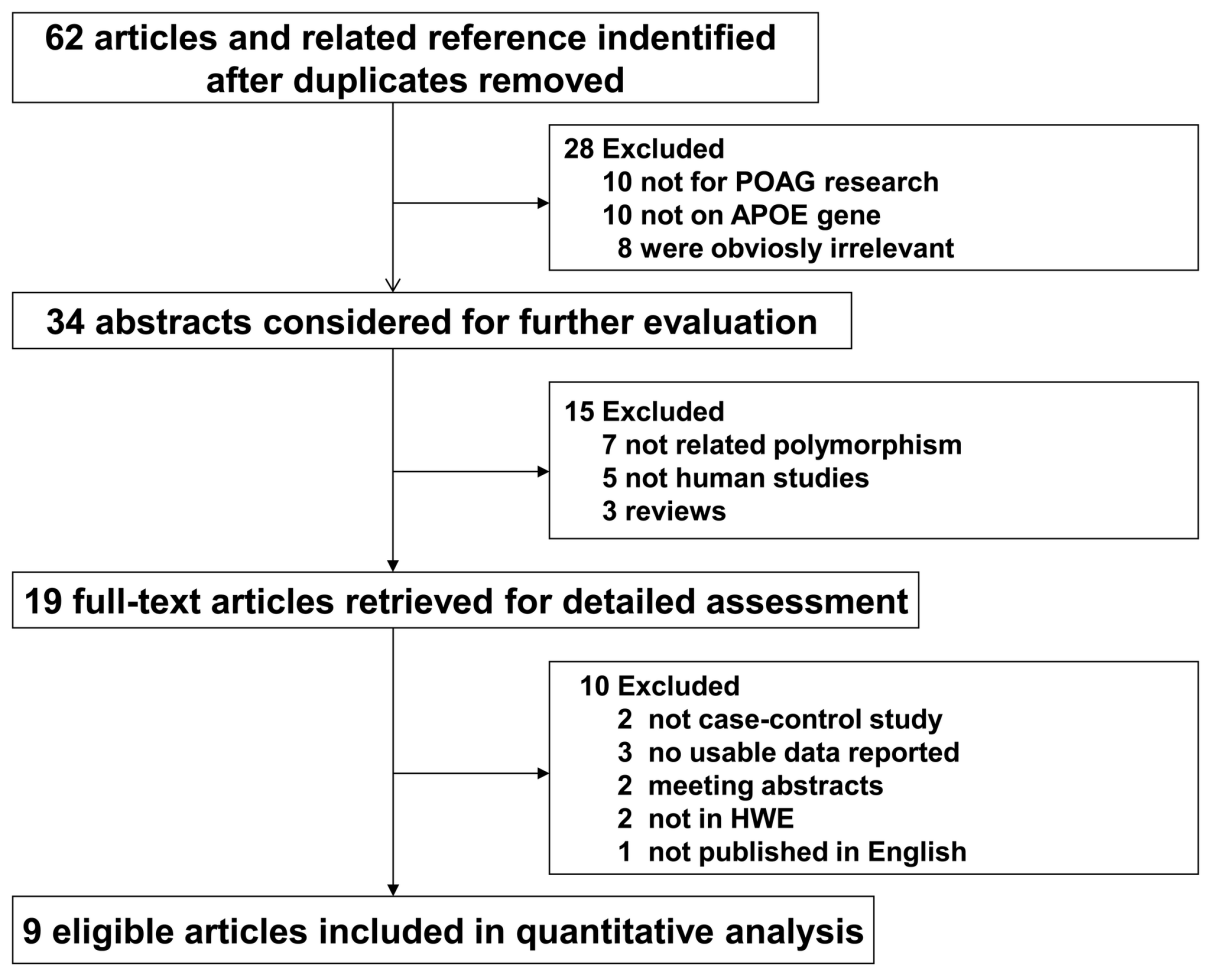
Flow diagram of the identification of relevant studies.

Our initial search generated 62 potentially relevant articles in the PubMed and Web of Science databases. Based on titles and the contents of the abstracts, 43 articles were excluded. Of the remaining 19 articles, two were not case-control studies, three did not report usable reported data, two were meeting abstracts, one was not published in English, and two studies were not in HWE; these studies were also excluded. In total, nine studies of the APOE ε2/ε3/ε4 polymorphism and its influence on POAG risk were included in the current meta-analysis based on our inclusion/exclusion criteria [28-36]. The literature search and study selection procedures are shown in Figure 1. These studies were published between 2002 and 2009. All studies were case-control studies and included three studies on Caucasians, five of Asians and one of Tasmanians, who were categorized into the other group. Eight studies had larger sample sizes (numbers of cases or controls>50), and only one study had a small sample size (i.e., the numbers of cases or controls<50). The genotype distributions of the controls of all studies were consistent with the Hardy-Weinberg equilibrium. The detailed characteristics of the eligible studies are shown in Table 1.

**Table 1:** Characteristics of the individual studies included in the meta-analysis.

**First author**	**Year**	**Country**	**Ethnicity**	**Sample size**		**Cases**						**Controls**					
				**Cases**	**Controls**	**ε2/2**	**ε2/3**	**ε2/4**	**ε3/3**	**ε3/4**	**ε4/4**	**ε2/2**	**ε2/3**	**ε2/4**	**ε3/3**	**ε3/4**	**ε4/4**
Vickers	2002	Australia	Other	142	51	6	8	7	78	42	1	2	9	2	30	6	2
Lake	2004	UK	Caucasian	155	349	1	16	10	91	31	6	3	37	13	208	81	7
Mabuchi	2005	Japan	Asian	310	179	0	14	2	259	35	0	0	18	0	123	38	0
Fan	2005	China	Asian	400	281	0	74	5	280	40	1	0	40	8	189	44	0
Lam	2006	China	Asian	400	300	0	74	5	280	40	1	0	42	8	203	47	0
Tamura	2006	Japan	Asian	28	77	0	2	1	16	8	1	2	7	1	61	6	0
Zetterberg	2007	Sweden	Caucasian	242	187	1	42	6	145	44	4	2	34	4	110	35	2
Saglar	2009	Turkey	Caucasian	75	119	0	12	1	53	8	1	0	9	1	88	19	2
Jia	2009	China	Asian	176	200	2	25	5	112	29	3	1	29	4	136	28	2

### Main meta-analysis results

**Figure 2:**
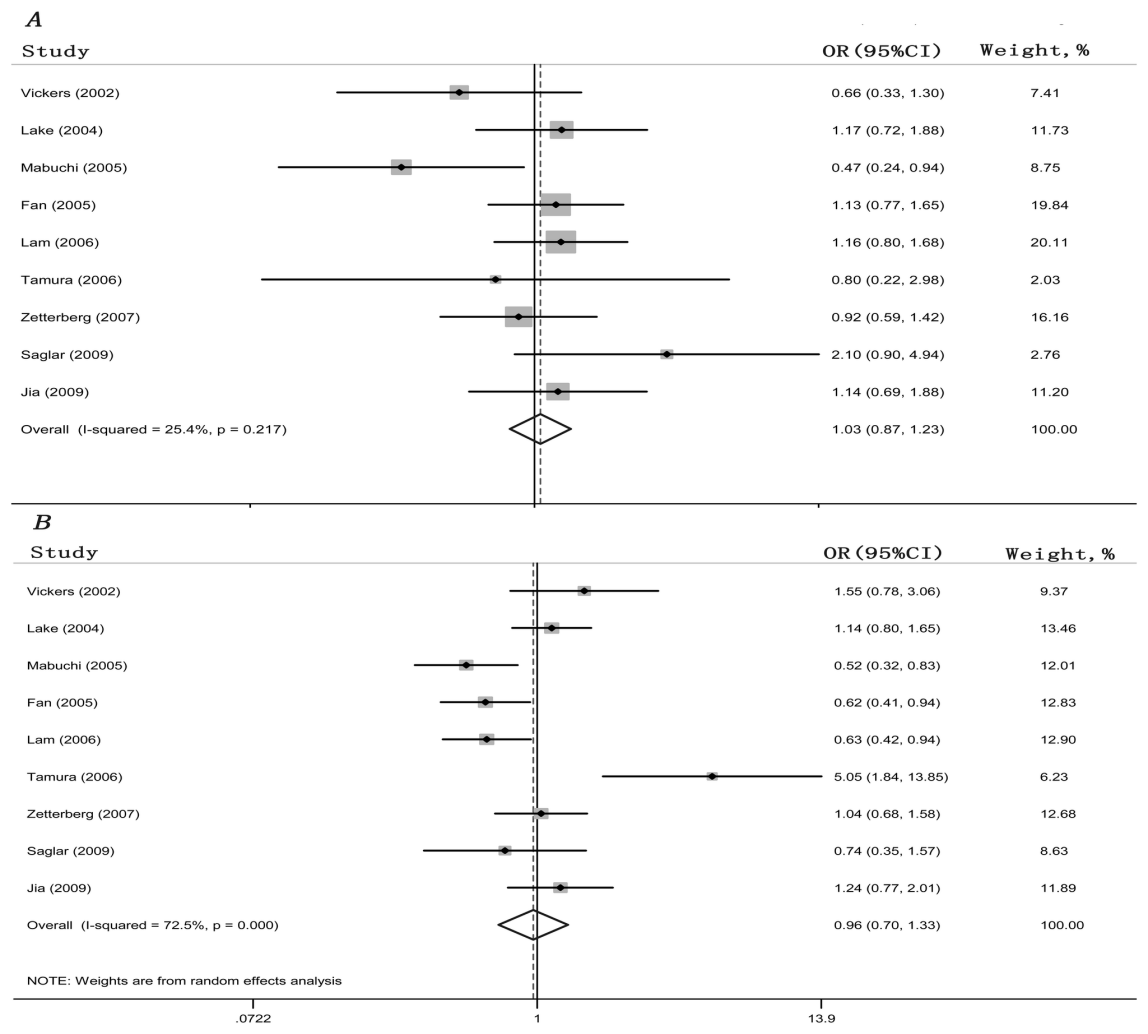
Forest plot of the association between the APOE ε2/ε3/ε4 polymorphism and POAG risk. Each study is shown by the point estimate of the OR with the 95% CI. (A) ε2 vs ε3, fixed-effects model. (B) ε4 versus ε3, random-effects model.

Table 2 lists the main results of this meta-analysis. A total of 1928 patients with POAG and 1793 unrelated matched controls from the nine studies were included in this meta-analysis. Overall, no significant main effects on POAG susceptibility were observed in the overall population in any of the allelic comparison models (ε2 vs ε3: OR = 1.034, 95% CI = 0.871-1.227; ε4 vs ε3: OR = 1.045, 95% CI = 0.807-1.353) or genotypic comparison models (ε2/2 vs ε3/3: OR = 0.950, 95% CI = 0.482-1.871; ε2/3 vs ε3/3: OR = 0.964, 95% CI = 0.706-1.317; ε4/4 vs ε3/3: OR = 1.339, 95% CI = 0.763-2.350; ε3/4 vs ε3/3: OR = 0.917, 95% CI = 0.637-1.321; ε2/2 + ε2/3 vs ε3/3: OR = 1.025, 95% CI = 0.842-1.248; ε2/2 vs ε3/3+ ε2/3: OR = 0.979, 95% CI = 0.499-1.920; ε3/4 + ε4/4 vs ε3/3: OR = 0.938, 95% CI =0.655-1.342; ε4/4 vs ε3/3 + ε3/4: OR = 1.325, 95% CI = 0.757-2.321). Figure 2A and 2B illustrate the overall meta-analysis of the APOE ε2/ε3/ε4 polymorphism and POAG risk for the allelic comparison models.

**Table 2:** Determination of the genetic effect of APOE ε2/ε3/ε4 polymorphism on POAG and subgroup analyses.

Genetic Contrasts	Comparisons	Studies (n)	Heterogeneity test		Model selected	OR(95%CI)
			P value	I^2^		
ε2 vs ε3	Overall	9	0.217	25.4	Fixed	1.034 (0.871,1.227)
	Caucasian	3	0.23	32	Fixed	1.118 (0.827,1.513)
	Asian	5	0.213	31.3	Fixed	1.038 (0.835,1.290)
ε4 vs ε3	Overall	9	0	72.5	Random	0.965(0.700,1.329)
	Caucasian	3	0.592	0	Fixed	1.045(0.807,1.353)
	Asian	5	0	81.6	Random	0.913(0.540,1.544)
ε2/2 vs ε3/3	Overall	9	0.992	0	Fixed	0.950(0.482,1.871)
	Caucasian	3	0.727	0	Fixed	0.870(0.280,2.705)
	Asian	5	0.932	0	Fixed	1.046(0.363,3.018)
ε2/3 vs ε3/3	Overall	9	0.037	51.3	Random	0.964(0.706,1.317)
	Caucasian	3	0.265	24.7	Fixed	1.092(0.758,1.574)
	Asian	5	0.053	57.1	Fixed	1.060(0.830,1.355)
ε2/2 + ε2/3 vs ε3/3	Overall	9	0.059	46.6	Fixed	1.025(0.842,1.248)
	Caucasian	3	0.24	30	Fixed	1.063(0.742,1.523)
	Asian	5	0.052	57.5	Fixed	1.062(0.832,1.356)
ε2/2 vs ε3/3 + ε2/3	Overall	9	0.994	0	Fixed	0.979(0.499,1.920)
	Caucasian	3	0.761	0	Fixed	0.858(0.276,2.664)
	Asian	5	0.94	0	Fixed	1.028(0.358,2.955)
ε4/4 vs ε3/3	Overall	9	0.641	0	Fixed	1.339(0.763,2.350)
	Caucasian	3	0.807	0	Fixed	1.561(0.723,3.375)
	Asian	5	0.698	0	Fixed	1.663(0.642,4.309)
ε3/4 vs ε3/3	Overall	9	0.001	69.7	Random	0.917(0.637,1.321)
	Caucasian	3	0.839	0	Fixed	0.879(0.635,1.215)
	Asian	5	0.001	78.4	Random	0.852(0.491,1.476)
ε3/4 + ε4/4 vs ε3/3	Overall	9	0.001	70.4	Random	0.938(0.655,1.342)
	Caucasian	3	0.799	0	Fixed	0.930(0.681,1.270)
	Asian	5	0	80.6	Random	0.893(0.503,1.586)
ε4/4 vs ε3/3 + ε3/4	Overall	9	0.621	0	Fixed	1.325(0.757,2.321)
	Caucasian	3	0.805	0	Fixed	1.604(0.745,3.453)
	Asian	5	0.834	0	Fixed	1.637(0.632,4.236)

OR, odds ratio; CI, confidence interval.

We also performed subgroup analyses that were stratified by ethnicity. Overall, no obvious evidence of associations between the APOE ε2/ε3/ε4 variants and the risk of POAG were found in any Asian or Caucasian genetic model. The results of these analyses are shown in Table 2.

### Test for heterogeneity

There was significant heterogeneity in the following four genetic models: ε4 vs ε3: P_heterogeneity_ < 0.001; ε2/3 vs ε3/3: P_heterogeneity_ =0.037; ε3/4 vs ε3/3: P_heterogeneity_ = 0.001; and ε3/4 + ε4/4 vs ε3/3: P_heterogeneity_ =0.001. When patients were stratified based on ethnicity, the heterogeneity of the Caucasian samples disappeared in the following four genetic models: ε4 vs ε3: P_heterogeneity_ = 0.592; ε2/3 vs ε3/3: P_heterogeneity_ = 0.265; ε3/4 vs ε3/3: P_heterogeneity_ = 0.839; and ε3/4 + ε4/4 vsε3/3: Pheterogeneity = 0.799. Data of heterogeneity test are listed in Table 2.

### Sensitivity Analyses

**Figure 3:**
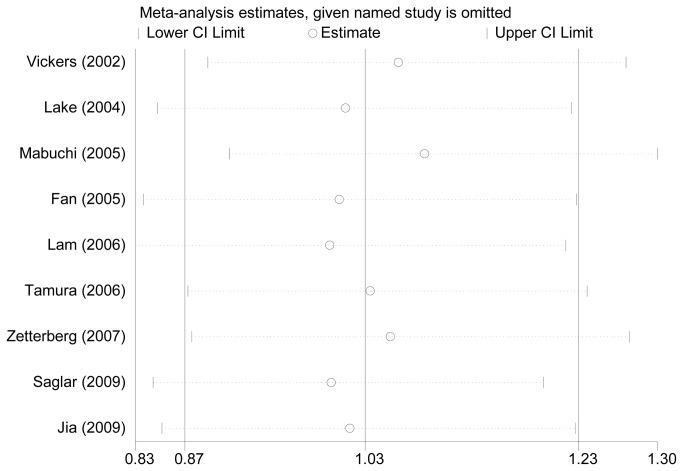
Sensitivity analyses through deletion of one study at a time to reflect the influence of the individual dataset to the pooled ORs.

Sensitivity analyses were performed after sequential removal of each of the included studies to examine the influence of each individual data-set on the pooled ORs. No single study qualitatively changed the pooled ORs, which indicates that the results of this meta-analysis were basically stable and robust (Figure 3).

### Publication Bias Diagnostics

**Figure 4:**
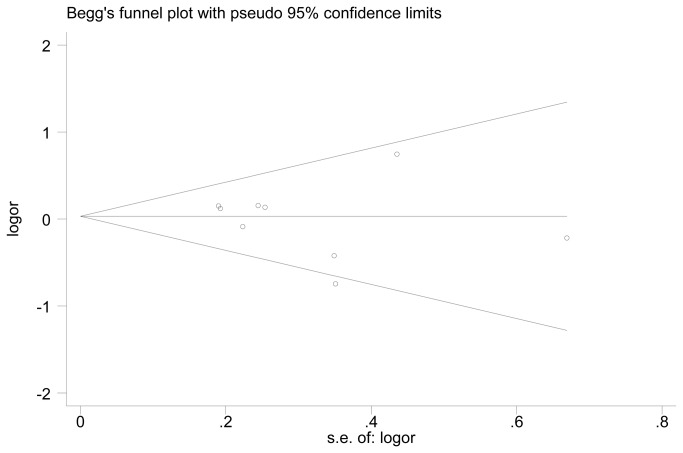
Funnel plot for the publication bias test in the meta-analysis investigating the association between the APOE ε2/ε3/ε4 polymorphism and POAG risk.

Funnel plots and Egger’s tests were used to assess potential publication biases of the literatures. The shapes of the funnel plots did not reveal any evidence of obvious asymmetries (Figure 4). Similarly, the results of Egger’s tests did not show significant publication biases in the current meta-analysis (t = -0.70, P = 0.504 for ε2 vs ε3 and t = 1.51, P = 0.176 for ε4 vs ε3).

## Discussion

Primary open-angle glaucoma (POAG) is the most frequent type of glaucoma and one of main causes of irreversible blindness worldwide [37]. POAG is the most common form of glaucoma in Europe, Africa, South Asia, and Latin America, and the reported prevalence rates range from 1.1% to 3.8% [38-40]. POAG is a complex disease in which many factors, including environmental factors, genetic alterations and their combined interactions are involved. Genetic association studies of POAG that have been conducted in the recent years, particularly genome-wide association studies (GWAS), have been extremely successful and have identified several genetic loci that are associated with disease susceptibility, which have provided the opportunity to take a fresh look at the genetic factors involved in POAG [41-43]. 

APOE is a 36-kDa glycoprotein that plays an essential role in lipid and cholesterol transport [13,14]. The APOE gene plays an important role in the development of Alzheimer’s disease (AD). There is strong evidence that the prevalence of POAG is greater in AD patients, and an association between POAG and Alzheimer’s disease exists [44,45]. It has also been reported that AD and glaucoma share some common features and that AD patients exhibit widespread axonal degeneration of the optic nerves and the loss of retinal cells, especially ganglion cells [46,47]. Furthermore, research has revealed that similar neurofilament triplet proteins are susceptible to neurofibrillary tangle formation in AD and POAG at the cellular level [48]. Xin et al conducted a meta-analysis in 2010 and found that APOE polymorphisms were significantly associated with the development AD [49]. In the last decade, the focus on genetic susceptibilities to POAG has led to increased attention on the study of the gene polymorphisms involved in the pathogenesis of POAG. A number of studies have investigated the association between the APOE ε2/ε3/ε4 polymorphism and POAG susceptibility, but the results of these studies are contradictory. To derive a more precise estimation of this relationship, we performed large meta-analysis.

To our knowledge, this is the first meta-analysis of published studies that has investigated whether the APOE ε2/ε3/ε4 polymorphism is associated with the risk for POAG. In this study, nine eligible studies comprising 1928 patients with POAG and 1793 unrelated matched controls were included. Overall, we found no obvious evidence for an association between the APOE ε2/ε3/ε4 polymorphism and the risk for POAG in any allelic or genotypic model. Furthermore, analyses of subgroups created based on ethnicity also failed to reveal any significant association of this polymorphism and POAG susceptibility.

Heterogeneity is a potential problem that might affect the interpretation of our results. In our meta-analysis, significant heterogeneity was found for the following genetic models:ε4 vs ε3, ε2/3 vs ε3/3, ε3/4 vs ε3/3 and ε3/4 + ε4/4 vs ε3/3. However, in the subgroup analyses stratified by ethnicity, the heterogeneity disappeared among Caucasians in these four genetic models. This heterogeneity may have resulted from differences in the patients’ genetic backgrounds, living environments or genotyping methods. Moreover, the leave-one-out sensitivity analyses revealed that no single study influenced the overall results qualitatively, which indicates that our results are reliable. Additionally, neither the shapes of the funnel plots nor the statistical results revealed publication biases in our meta-analysis.

To some extent, some limitations of the current meta-analysis should be acknowledged when interpreting the results. First, we only included published data from the selected databases; it is possible that some relevant published studies or unpublished studies were missed, and this may have biased the results. Second, due to the lack of POAG subtype information from the eligible studies, we were unable to perform sub-group analyses based on POAG types, (e.g., high-tension glaucoma or normal-tension glaucoma), which play a crucial roles in genetic analyses. Third, due to the limited availability of published results, the number of publications included in our meta-analysis was only seven; this low number is bound to bias the analyses results. We expect that more studies with larger samples and randomized -controlled trials will become available. Finally, only papers that were published in English and studies with available full-text articles were included in the current meta-analysis; therefor, some eligible studies that have not been unpublished or were reported in other languages were missed, which may influence the pooled results. Despite these limitations, our meta-analysis also has some advantages. First, we significantly increased the statistical power of our meta-analysis by including a substantial number of cases and controls from different studies. Second, the methodological issues of meta-analysis, such as heterogeneity, publication bias, and the stability of results were all carefully investigated. Third, the qualities of the case-control studies identified in our meta-analysis were satisfactory and met our inclusion criteria.

In summary, this meta-analysis provided evidence that the APOE ε2/ε3/ε4 polymorphism is not associated with POAG susceptibility. Well-designed case-control studies with adequate numbers of cases are warranted to confirm our findings.

## Supporting Information

Checklist S1 (DOC)
